# Combined applications of fine needle aspiration cytology and Flow cytometric immunphenotyping for diagnosis and classification of non Hodgkin Lymphoma

**DOI:** 10.1186/1742-6413-3-24

**Published:** 2006-10-27

**Authors:** Pranab Dey, Thasneem Amir, Aisha Al Jassar, Salem Al Shemmari, Sanjay Jogai, Ganapathi Bhat M, Aisha Al Quallaf, Zahia Al Shammari

**Affiliations:** 1 Cytology Department, Kuwait Cancer Control Center, Suwaikh, Kuwait; 2 Department of cytology, Post Graduate Institute of Medical Education and Research, Chandigarh, India; 3 Haematology Department, Kuwait Cancer Control Center, Suwaikh, Kuwait; 4 Flow cytometry Laboratory, Kuwait Cancer Control Center, Suwaikh, Kuwait

## Abstract

**Aims and objectives:**

In this present study we have evaluated the feasibility of sub-classification of non-Hodgkin's lymphoma (NHL) cases according to World Health Organization's (WHO) classification on fine needle aspiration cytology (FNAC) material along with flow cytometric immunotyping (FCI) as an adjunct.

**Materials and methods:**

In this five years study, only cases suggested or confirmed as NHL by FNAC were selected and FCI was performed with a complete panel of antibodies (CD3, CD2, CD 4, CD5, CD8, CD7, CD10, CD19, CD20, CD23, CD45, κ and λ) by dual color flow cytometry. Both cytologic findings and FCI data were interpreted together to diagnose and sub-classify NHL according to WHO classification. Wherever possible the diagnoses were compared with cytology.

**Results:**

There were total 48 cases included in this study. The cases were classified on FNAC as predominant small cells (12), mixed small and large cells (5) and large cells (26). In five cases a suggestion of NHL was offered on FNAC material and these cases were labeled as NHL not otherwise specified (NHL-NOS). Flow cytometry could be performed in 45 cases (93.8%) and in rest of the three cases the material was inadequate because of scanty blood mixed aspirate. Light chain restriction was demonstrated in 30 cases out of 40 cases of B-NHL (75%). There were 15 cases each of κ and λ light chain restriction in these 30 cases. With the help of combined FCI and FNAC, it was possible to sub-classify 38 cases of NHL (79%) according to WHO classification. Combined FNAC and FCI data helped to diagnose 9 cases of small lymphocytic lymphoma (SLL), 2 cases of mantle cell lymphoma (MCL), 4 cases of follicular lymphoma (FL), 17 cases of diffuse large B lymphoma (DLBL) and 6 cases of lymphoblastic lymphoma. Histopathology diagnosis was available in 31 cases of NHL out of which there were 14 recurrent and 17 cases of primary NHL. Out of 15 DLBL cases diagnosed on FCI and FNAC, histology confirmed 14 cases and one of these cases was diagnosed as Burkitt's lymphoma on histology. Cases of FL (4), SLL (3) and MCL (2) were well correlated with histopathology. Out of the five cases suggestive of NHL on cytology, histopathology was available in four cases. Histology diagnosis was given as DLBL (1), SLL (1), anaplastic large cell lymphoma (1) and FL transformed into large cell NHL (1). Considering histopathology as gold standard, diagnostic specificity of combined FNAC and FCI was 100% (31/31) and sensitivity in sub-classification was 83.8% (26/31).

**Conclusion:**

FNAC combined with FCI may be helpful in accurately sub-classifying NHL according to WHO classification. Many of the subtypes of NHL such as FL and MCL which were previously recognized as a pure morphologic entity can be diagnosed by combined use of FNAC and FCI. Other ancillary investigations such as chromosomal changes, cell proliferation markers etc. may be helpful in this aspect.

## Background

Fine needle aspiration cytology (FNAC) is a very helpful technique in diagnosis of benign and malignant lesions of lymph node [1-4]. Many authors also claim that FNAC can accurately diagnose Hodgkin's and non-Hodgkin's lymphoma (NHL)[5,6]. However there is a wide variation of diagnostic sensitivity and specificity of FNAC in non-Hodgkin's lymphoma [5-8]. The role of cytology in primary diagnosis and sub-classification of non-Hodgkin's lymphoma is controversial [9-12]. After the introduction of REAL/WHO classification, there is much difference in the cytologist's approach of lymphoma diagnosis and classification. WHO and REAL classification emphasized immense importance on the cytomorphology and immunophenotype of lymphoma for accurate sub classification [13,14]. In this present study we have analyzed the role of flow cytometric immunotyping as an adjunct to FNAC for diagnosis and sub-classification of NHL according to WHO classification.

## Materials and methods

This study is of five years duration from the year 2000 January to 2004 December. Only cases suggested or confirmed as NHL by FNAC were selected. FNAC smears were prepared for May Grunwald Giemsa (MGG) and Haematoxyline and Eosin stain in each case. The May Grunwald Giemsa smears were studied immediately. A second pass of the needle was done and material was collected in citrate buffer for flow cytometric immunophenotyping (FCI). The specimen was immediately processed and a complete panel of antibodies was used for immunophenotyping. Both cytologic findings and FCI data were interpreted together to diagnose and sub-classify NHL according to WHO classification as far as possible. Wherever possible the final histological diagnosis was correlated with FNAC and FCI diagnosis.

### Specimen preparation

FNAC material was collected in citrate buffer solution and immediately transferred to flow cytometry laboratory. The sample was washed in phosphate buffer solution three times for 5 minutes at 2000 revolutions per minute. The supernatant fluid was discarded and the deposits of cells were studied for cell viability and count. After that, the suspension was divided into multiple tubes depending on the adequacy of the cell. Samples were then incubated for 15 minutes in dark with 5 μl of antibody solution tagged with Fluorescein isothyocyanate (FITC) or Phycoerythrin (PE). The following antibodies were used: CD3, CD2, CD 4, CD5, CD8, CD7, CD10, CD19, CD20, CD23, CD45, κ and λ. (Becton Dicikinson, San Jose, CA, USA). After incubation, red blood cells were lysed by a lysing solution (Becton Dicikinson, USA, Catalogue no 349202) for 15 minutes and then washed by a cell wash solution (Becton Dicikinson, USA, Catalogue no 349524). After centrifuging, the supernatant solution was discarded and 500 μl cell fixative was added (Becton Dicikinson, Catalogue no 340181). Flow cytometry analysis was then performed in Becton Dicikinson flow cytometer (San Jose, CA, USA) by dual-color analysis technique. Gating was done using forward and side scatter and also by the help of Cell Quest software (San Jose, CA, USA). We analyzed a minimum number of 10,000 events for each cell marker.

## Results

**Figure 1:**
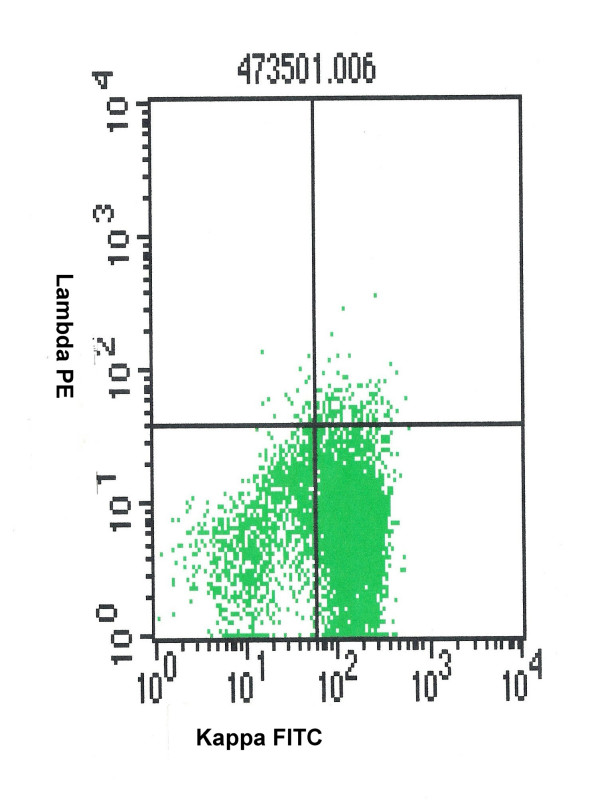
kappa light chain restriction in a case of B-non Hodgkin's lymphoma.

There were total 48 cases included in this study and in all these cases FNAC confirmed or suggested the diagnosis of NHL. Age of the patients ranged from 6 year to 78 years. There were 34 male and 14 female patients. Twenty-nine cases were first time investigated in our FNAC clinic for primary diagnosis and 19 cases were recurrent NHL. In 36 cases, the masses were palpable and FNAC was done without any radiological guidance (table 1). In 12 cases masses were non-palpable and FNAC was done with the help of Ultrasonographic guidance. FNAC yielded adequate material in all these deep-seated cases. The cases were classified on FNAC as predominant small cells (12), mixed small and large cells (5) and large cells (26) [table 2]. In five cases a suggestion of NHL was offered on FNAC material and these cases were labeled as NHL not otherwise specified (NHL-NOS). Flow cytometry could be performed in 45 cases (93.8%) and in rest of the three cases the material was inadequate because of scanty blood mixed aspirate. There were total 40 cases of B-NHL. These cases showed predominant CD19 and CD20 positive cell population. Light chain restriction (figure 1) could be demonstrated in 30 out of 40 cases of B-NHL (75%). There were 15 each cases of κ and λ light chain restriction. In 10 cases there was no demonstrable light chain restriction on FCI. However cytology smears and predominant population of CD19 cells indicated a NHL of B cell origin.

**Table 1:** Anatomic distribution of aspirated lesions

**Sites**	**Number of patients**
Palpable	
Cervical lymph node	19
Inguinal lymph node	3
Axillary lymph node	8
Submandibular lymph node	3
Supraclavicular lymph node	2
Skin	1
Ultra Sonogram guided	
Abdominal lymph node	6
Bowel wall	2
Mediastinum	3
Testis	1

**Table 2:** Cytology and flow cytometry, diagnosis

**FNAC**	**FCI**							
	
	**DLBL**	**FL**	**SLL**	**MCL**	**T-lymphoblastic NHL**	**B-lymphoblastic NHL**	**NHL-unclassified**	**No opinion**
Small cell 12		1	8	2				1
Mixed cell 5		3					2	
Large cell 26	16				5	1	3	1
Suggestive of NHL 5	1		1				2	1

DLBL = Diffuse large B lymphoma, SLL = Small lymphocytic lymphoma, FL = Follicular lymphoma, MCL = Mantle cell lymphoma, FNAC = Fine needle aspiraition cytology, NHL = Non Hodgkin's lymphoma, FCI = Flow immunophenotyping

**Figure 2:**
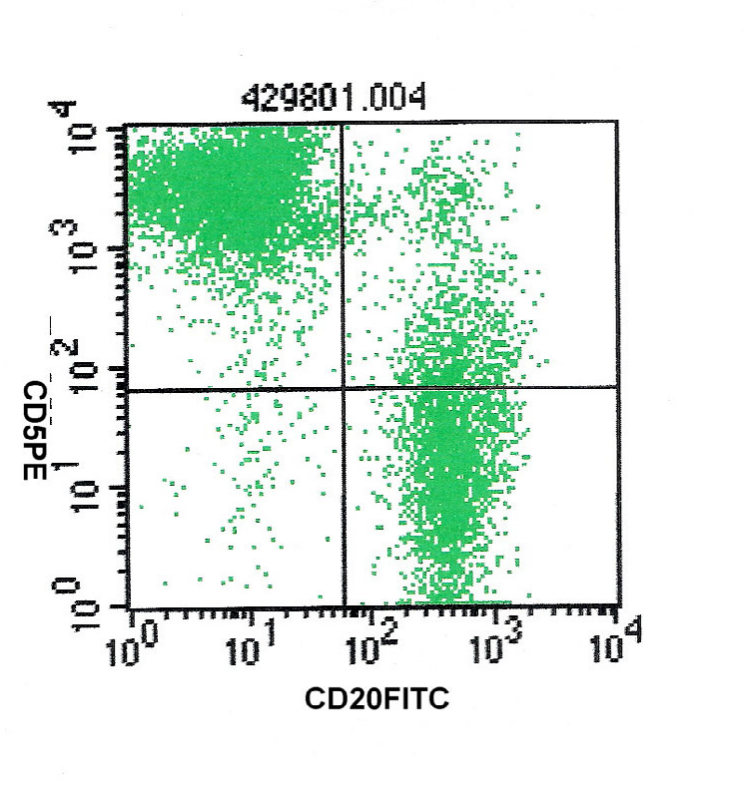
CD5 and CD20 positivity in a case of small lymphocytic lymphoma.

**Figure 3:**
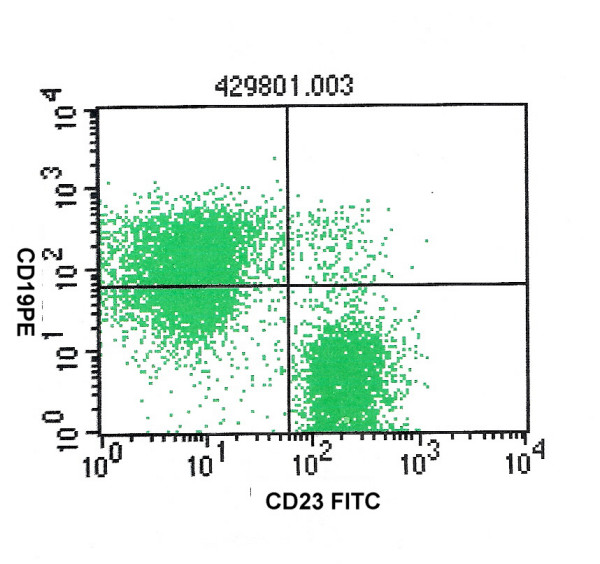
CD23 and CD19 positivity in that same case of small lymphocytic lymphoma.

**Figure 4:**
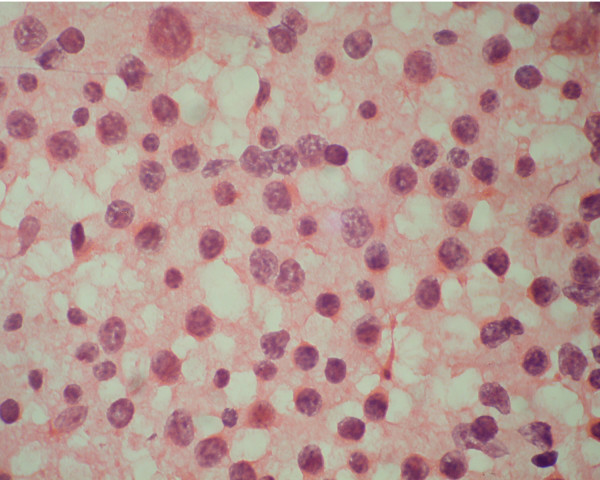
Cytology smear of small Lymphocytic lymphoma showed small cells with clumped chromatin. (Haematoxylene and Eosin stain).

The diagnostic distribution of cases on the basis of FCI is highlighted in table 2. With the help of combined FCI and FNAC, it was possible to sub-classify 38 cases of NHL (79%) according to WHO classification (table 3). Out of the 12 cases of small cell NHL, with the help of FCI we were able to diagnose a total of 8 cases of small lymphocytic lymphoma (SLL), 2 cases of mantle cell lymphoma (MCL) and one case of follicular lymphoma (FL). Due to inadequate material, no opinion was possible on FCI in one case of NHL. The SLL cases showed CD 19 and CD 20 positive cell population along with expression of CD5 and CD23 (figure 2, 3). Individual cells show round to oval small nuclei and clumped chromatin with no nucleoli (figure 4). In contrast, MCL lymphoma showed CD 19 and CD 20 positive cell population along with CD5 positive and CD23 negative marker. However on FNAC smears it was impossible to distinguish SLL and MCL by cytology alone.

**Table 3:** Combined Cytology and flow cytometry diagnosis

**Cytology and flow cytometry diagnosis**	**Number of cases**
FL	4
SLL	9
MCL	2
DLBL	17
Lymphoblastic (B)	1
Lymphoblastic (T)	5
NHL, unclassified	7
No opinion on FCI, but diagnosed as NHL on FNAC	3

DLBL = Diffuse large B lymphoma, SLL = Small lymphocytic lymphoma, FL = Follicular lymphoma, MCL = Mantle cell lymphoma, FNAC = Fine needle aspiration cytology, NHL = Non Hodgkin's lymphoma, FCI = Flow immunophenotyping

**Figure 5:**
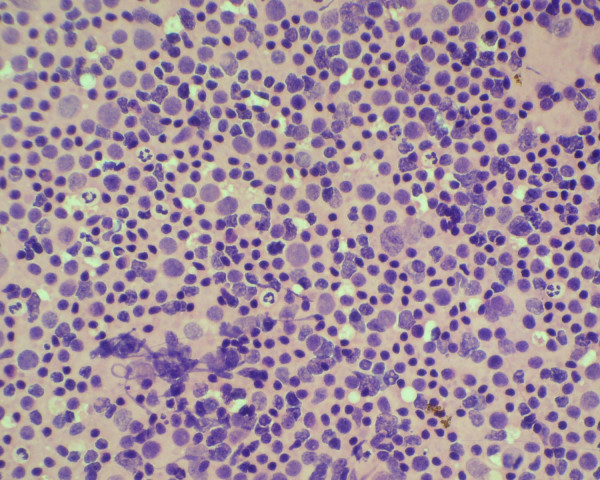
Cytology smear of a case of follicular lymphoma showing mixed small and large cells. (Haematoxylene and Eosin stain).

There were 5 cases of mixed small and large cell NHL, and out of which three cases were diagnosed as follicular lymphoma (FL) and two cases remained unclassified even after FCI as these cases did not show CD 10 positivity on FCI. The cases of FL showed CD10 positive cell population along with CD19 and CD20 positivity. Cytology smears showed mixed population of small and large round cells with scanty cytoplasm. Individual cells had round nuclei, fine chromatin and inconspicuous nucleoli (figure 5).

**Figure 6:**
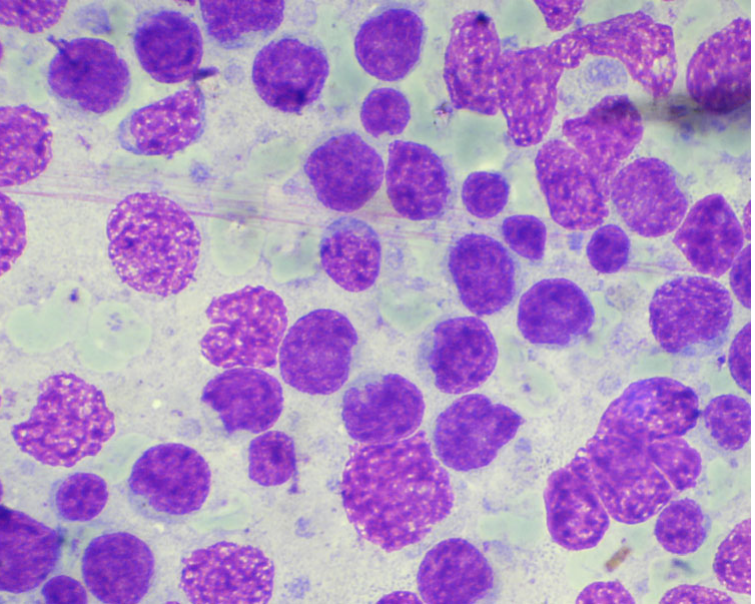
Aspiration cytology smear of a diffuse large B cell lymphoma showing large lymphoid cells with fine chromatin and occasional prominent nucleoli. (May Grunwald Giemsa stain).

**Figure 7:**
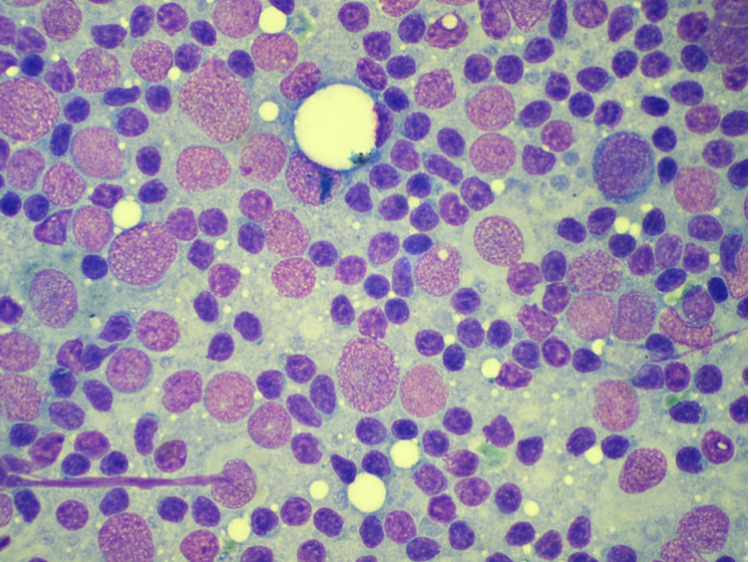
Aspiration cytology smear of a case of lymphoblastic lymphoma showing large cell with reticular chromatin and inconspicuous nucleoli. (May Grunwald Giemsa stain).

Out of 26 cases of large cell NHL, there were 16 cases of diffuse large B lymphoma (DLBL), 6 cases of lymphoblastic lymphoma and three cases remained unclassified. No opinion could be given on FCI in one case. DLBL cases showed B cell markers positivity (CD 19 and CD 20 positive cell population). Cytology smears of these cases showed monomorphic population of large cells with fine nuclear chromatin and occasional prominent nucleoli (figure 6). There were six cases of lymphoblastc lymphoma on cytology smears and FCI showed B cell marker positivity in one cases and T cell marker positivity in five cases. The cytology smears of these cases showed dissociated large cells with reticular chromatin and inconspicuous nucleoli (figure 7).

**Figure 8:**
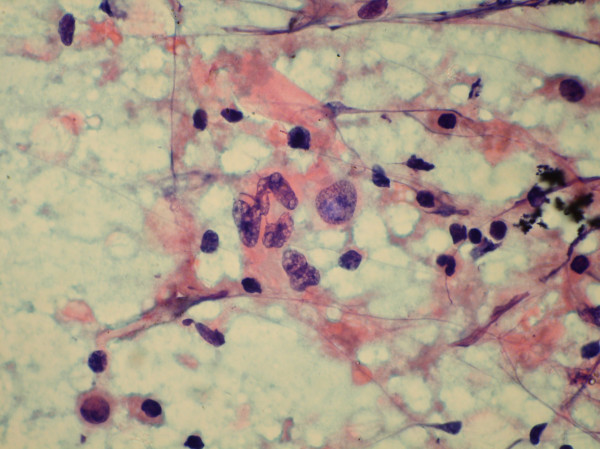
Cytology smear of a case of anaplastic large cell lymphoma showing dissociated cells with moderately pleomorphic large nuclei. (Haematoxylene and Eosin stain).

Five cases were diagnosed as suggestive of NHL. With the help of FCI, Sub classification was possible only in two cases (one each case of DLBL and SLL). In two other cases, FCI just confirmed the presence of lymphomas. One of these two cases was finally diagnosed as FL transformed into blastic phase on histopathology. The other case was labeled as lymphoma (not otherwise specified). This case showed predominant CD 19 and CD 20 positivity (80 and 82%) and κ chain expression only. No other marker was positive to help in subclassification of this lymphoma. Histopathology of this case was also not available. In the fifth case, FCI was inadequate for any opinion and this case was finally diagnosed on histopathology as anaplastic large cell lymphomas. Cytology smear of this case showed dissociated large atypical cells with mild to moderate nuclear pleomorphism having prominent nucleoli (figure 8).

Histopathology diagnosis was available in 31 cases of NHL and out of which there were 14 recurrent and 17 primary NHL. Table 4 shows the correlation between combined FNAC and FCI diagnosis versus histology diagnosis. Out of 15 DLBL cases on FCI and FNAC, histology confirmed 14 cases. One case was diagnosed as Burkitt's lymphoma. Cases of FL (4), SLL (3) and MCL (2) were well correlated with histopathology. Out of the five cases of suggestive of NHL on cytology, histopathology was available in four cases. Histopathology of these cases showed DLBL (1) and SLL (1), anaplastic large cell lymphoma (1) and FL transformed into large cell NHL (1). Diagnostic specificity of combined FNAC and FCI was 100% (31/31) and sensitivity in sub-classification was 83.8% (26/31) in accordance with histopathology.

**Table 4:** Combined FNAC and FCI versus available histopathology diagnosis

**Combined FNAC and FCI diagnosis**	**Histology diagnosis**					
	
	**DLBL**	**FL**	**SLL**	**MCL**	**Lymphob lastic**	**Other**
DLBL (15)	14					1 (Burkitt's)
FL (4)		4				
SLL (3)			3			
MCL (2)				2		
Lymphoblastic (3)					3	
Suggestive of NHL (4)	1		1			1 ALCL 1 FL to large B-NHL

DLBL = Diffuse large B lymphoma, SLL = Small lymphocytic lymphoma, FL = Follicular lymphoma, MCL = Mantle cell lymphoma, ALCL = Anaplastic large cell lymphoma, FNAC = Fine needle aspiration cytology, NHL = Non Hodgkin's lymphoma, FCI = Flow immunophenotyping

## Discussion

FNAC has been widely used for the rapid diagnosis of metastatic malignancies, infectious diseases and reactive lymphoid hyperplasias [1-4]. However there is a wide divergence of opinions regarding the role of FNAC on primary diagnosis and sub-classification of NHL on FNAC smears [9-12]. This difficulty is particularly problematic when a less than pure monomorphic lymphocyte population exists in the smears. A major disadvantage inherent in the FNAC smear is the inability to identify a follicular growth pattern [15], which is a major prognostic factor. After the introduction of REAL and WHO classification, the major emphasis has been given on cell morphology and immunopheotype rather than the growth pattern [13,14]. In the present paper, we attempted to apply the combined approach of FNAC and FCI to implement WHO classification on FNAC. FCI showed light chain restriction in 75% (30/40) cases of B-NHL. In rest of the ten cases, there were predominant CD19 and CD20 positive cell populations (>80%). Considering the cytomorphology and predominant CD 19 and CD20 expression, we considered these cases as B-NHL. The lack of expression of surface immunoglobulin light chains in B-NHL is an unusual phenomenon. However, it has been also noted by other authors [16,17]. Zardawi et al [17] encountered seven cases of B-NHL, which did not show any light chain restriction on FCI. Similarly Zeepa et al [16] noted 12 cases of B-NHL with no demonstrable light chain restriction. In fact, it has been suggested by them that CD20 positivity in excess of 85% is diagnostic of NHL in a cytological suspicious case of lymphoma [16].

In addition to providing clear, quantitative evidence of monclonality, FCI was helpful in subclassification of NHL in 79% (38/48) cases. It was particularly helpful in subclassification of low grade B-NHL. The FNAC of small lymphocytic lymphomas, mantle cell lymphomas and low grade follicular lymphomas are difficult to differentiate by cytomorphology alone. Combining CD 19 with other antibodies such as CD 5, CD10, CD 23 and FMC 7 are usually helpful to distinguish these lesions. In the present study, all the SLL cases, expressed both CD5 and CD 23 antigen. SLL cases usually show absence of expression of CD 10 and FMC 7. Typically they show a low proliferative index. In MCL, we noted only CD5 positive and CD23 negative population of cells. We did not have FMC 7 antibody in our laboratory for additional confirmation in MCL cases. MCL is associated with characteristic cytogenesis abnormality of t(11:14). This anomaly causes overexpression of BCL1 gene, which encodes cycin D1. So MCL are positive for cyclin D1 in addition to CD 5 positivity. This is an additional characteristic feature of MCL [18]. CD 10 expression along with other B cell markers (such as CD19/CD 20), was useful in diagnosis of FL. These cases were negative for CD5. However in two cases CD10 positivity was not demonstrated and a confident diagnosis of FL was not possible. The FL is associated with the charateristics t(14:18)(q32;21) translocation. This can be demonstrated on cytology smears with the help of fluorescent in situ hybridisation (FISH) [19]. This reciprocal translocation may lead to oberexpression of BCL2 protein [20]. Demonstration of BCL2 protein overexpression may be helpful in histology sections where architecture is preserved.

FCI alone was not very helpful in distinguishing DLBL, lymphoblastic lymphoma and Burkitt's lymphoma, and in those cases cytomorphology on the FNAC smears was important. Lymphoblastic lymphomas are positive for early markers such as TdT and CD34. B cells may also express pan B-cell makers such as CD20 and lack surface and sometimes cytoplasmic immunoglobulin. We did not have adequate standardization of TdT and so we relied on cytomrphology to diagnose lymphoblastic lymphomas. The T-lymphoblastic lymphomas showed variable positivity of CD3, CD2 and CD10 markers. Wheras B-lymphoblastic lymphomas showed varaiable expression of CD19, CD20 and CD10. Careful history and peripheral blood picture should always take into consideration as Acute myeloid leukaemia (AML-M0) may simulate similar picture. The Burkitt's lymphomas have characteristic cytomorphology. FNAC smears show dissociated cells with deep blue vacuolated cytoplasm, having round regular nuclei with prominent nucleoli and abundant tingible body macrophages in the background. High cell proliferation markers (Ki 67 index more than 99%) and t (8;14)(q24;q32) translocation are the characteristic features of Burkitt's lymphomas [13]. The Burkitt lymphomas always express pan – B cell markers and are positive for CD 1O and lack expression of early markers such as CD 34 and TdT.

In this study we applied FCI to diagnose and subclassify NHL on FNAC smears according to WHO classification. There are a few available studies in this aspect [16,21-23]. Zeppa P et al [16] in a similar study, were able to subclassify 70 cases of NHL among the 115 cases of NHL with small and medium sized cells. Expression and coexpression of different CD markers were helpful in their study for subtyping NHL. In a similar study, Siebert JD et al [21] were able to subtype 29/38 (76.3%) NHL with the help of FCI. They also highlighted that adjunctive FCI and FNAC is potentially practicable in a community hospital and can help direct lymphoma therapy. Mourad et al[22] also were able to subclassify large number of cases according to WHO subclassification with the help of FCI. Mayall et al [23] in a recent paper showed that FNAC along with FCI were very helpful to diagnose and subclassify B cell NHL. But FCI had litlle use for T cell NHL in their study. Our study was also comparable with the above mentioned studies as we were also able to subclassify 83% of histopathology proven cases. We also were able to subclassify the large number of small cell B-NHL. It was not very informative in large cell type of B-NHL. In these cases cytomorphologic feature are helpful in subclassification of NHL. FCI is also likely to miss difficult cases such as ALCL. Monoclonality was difficult to prove in T-NHL on FCI, however aberrant expression of T cell markers and predominant expression of CD4 or CD8 indicated T cell NHL.

Other ancillary techniques such as cell proliferation markers, molecular markers etc. may be helpful in identifying certain type of NHL [18,24]. FCI of marzinal zone lymphoma is not characteristic and demonstration of t(11: 18) (q21:21) may be required [25]. Similarly translocation of t(8: 14) is characteristics of Burkitt's lymphoma and it involves c-myc oncogene expression [26]. Anaplastic large cell lymphoma has specific t(2:5) translocation and ALK 1 positivity may be a helpful marker in this variety of NHL [27].

FCI is a rapid technique and it helped us to objectively asses the monoclonality of the cell population. With the help of FCI, we quantiatatively estimated the cell population expressing particular CD markers and this was very helpful for subclassification of NHL according to WHO classificartion. In most of our cases adequate number of cells were present in FNAC material. The demonstration of expression of large number of CD markers in the lymphoid cell population was possible with the help of FCI. There is one advantage of FCI over routine immunocytochemistry. With the help of FCI, it is possible to see the co-expression of different CD markers in a single cell. This is particularly helpful in distinguishing SLL and MCL.

In brief, FNAC combined with FCI may be helpful in accurately subclassifying NHL according to WHO classification. Many of the subtypes of NHL such as FL and MCL which were previously recognized as a pure morphologic entity can be diagnosed by combined use of FNAC and FCI. Other ancillary investigations such as chromosomal changes, cell proliferation markers etc. may be helpful in this aspect [18]. In future FNAC supported by FCI and other ancillary investigations may replace excision tissue biopsy for histopathology particularly in recurrent cases of NHL.

## Competing interests

The author(s) declare that they have no competing interests.

## Authors' contributions

PD: Conception, design, acquisition of data, analysis and interpretation of data; 2) drafting the manuscript and 3) final approval of the version to be published.

TA, AJ, SS, SJ, GB, AQ, ZS: Equal contributions in this paper related to data collections and manuscript preparations.
